# Work-Time Compositions of Physical Behaviors and Trajectories of Sick Leave Due to Musculoskeletal Pain

**DOI:** 10.3390/ijerph18041508

**Published:** 2021-02-05

**Authors:** David M. Hallman, Nidhi Gupta, Leticia Bergamin Januario, Andreas Holtermann

**Affiliations:** 1 Centre for Musculoskeletal Research, Department of Occupational Health Sciences and Psychology, University of Gävle, 801 76 Gävle, Sweden; leticia.januario@hig.se; 2 National Research Centre for the Working Environment, 2100 Copenhagen, Denmark; ngu@arbejdsmiljoforskning.dk (N.G.); aho@arbejdsmiljoforskning.dk (A.H.)

**Keywords:** compositional data analysis, sickness absence, physical activity, sitting

## Abstract

We aimed to investigate the association between work-time compositions of physical behavior and sick leave trajectories due to musculoskeletal pain over one year. We conducted a secondary analysis using the data of 981 workers in a Danish prospective cohort (DPHACTO 2012–2014). At baseline, we assessed physical behaviors (sitting, standing, light physical activity (LIPA), and moderate-to-vigorous physical activity (MVPA)) at work and during leisure, using accelerometers. Over 1 year follow-up, workers reported sick-leave days due to musculoskeletal pain at 4-week intervals. Four distinct trajectories of sick leave were previously identified in this cohort (“no sick leave”, “few days—increasing trajectory”, “some days—decreasing trajectory”, “some days—increasing trajectory”), and used as an outcome in multinomial regression models with work-time compositions as predictors, adjusted for compositions of behavior during leisure, age, sex, body mass index, and smoking habits. More time spent sitting relative to the other behaviors was negatively associated with the trajectory of few days—increasing sick leave (*p* = 0.004), while time in LIPA was positively associated with the trajectory of some days—increasing sick leave (*p* = 0.009). Standing and MVPA were not significantly associated with sick leave trajectories. In conclusion, work-time compositions with more sitting relative to the other behaviors had lower risk for an increasing trajectory of sick leave due to pain, while compositions with more LIPA had higher risk. This may have implications for prevention of pain-related sick leave in blue-collar workers.

## 1. Introduction

Musculoskeletal pain (MSP) is one of the leading causes of sick leave in the working population [1], and imposes a large economic burden for organizations and society [2]. Identifying the main occupational determinants of sick leave due to MSP is crucial for the prevention of sick leave in the workforce [3].

Sick leave due to MSP is of multifactorial origin and a variety of possible occupational determinants of future sick leave due to MSP have been identified—for example, high physical work demands and poor psychosocial working conditions [4,5,6,7,8,9].

Time spent in daily physical behaviors such as sitting, standing, and physical activity has profound physiological and psychological effects that may extend to the onset and progression of MSP [10,11,12] and the ability to remain at work [13,14,15,16]. Overall, very few studies have addressed physical behaviors at work in relation to sick leave specifically due to MSP [17]. This warrants further research to gain knowledge supporting the prevention of pain related to sick leave in the workforce and providing guidance for organizations and practitioners.

Research on physical behavior at work has mostly relied on self-reported measurements with poor accuracy and high risk of differential bias [18,19,20]. Thus, there is a need for studies using accurate device-worn measurements to determine the extent to which physical behaviors at work impact on sick leave due to MSP [21].

Time-use in physical behaviors is constrained and compositional, as all behaviors sum up to a whole (e.g., 100% or a complete 8 h working day). Consequently, changing time spent engaging in one behavior will always result in more or less time in other behaviors. For example, an effective intervention reducing excessive standing time at work will inevitably lead to more time in other behaviors (e.g., sitting and walking). The constrained, dependent nature of time spent in different behaviors occurring during a working day requires the adoption of special analytical procedures—so-called compositional data analysis (CoDA) [22]. CoDA essentially transforms time-use data into ratios to address relative information between behaviors (e.g., sitting/non-sitting) instead of absolute information like minutes per day [23]. This approach leads to unconstrained data that can be used in standard statistical modeling such as regression analysis, and allows an integrated interpretation of physical behavior data.

The duration and long-term trajectory of sick leave spells vary considerably between individuals, which may reflect sub-groups of workers with different patterns of sick leave [24,25]. In a previous study on this population, we identified four distinct trajectories on the basis of monthly measurements of self-reported days on sick leave over one year [4]. We found that most workers (76%) had no sick leave at baseline and 1-year follow-up, 19% had few days at baseline (with a mean of 0.5 days/month) and an increasing trajectory of sick leave over 1 year, 3% had some days at baseline (9 days/month) and a decreasing trajectory, and 2% had some days at baseline (4 days/month) and an increasing trajectory. Therefore, we used these four trajectories of sick leave due to MSP as an outcome in this study.

The aim of this study was to determine the association between work-time compositions of device-worn measurements of physical behaviors and trajectories of sick leave due to MSP in workers.

## 2. Materials and Methods

### 2.1. Design and Study Sample

This is a secondary analysis of prospective data from the Danish Physical Activity Cohort with Objective measurements (DPHACTO). The study protocol and characteristics of DPHACTO were described previously [21,26]. The study population consists of blue-collar workers from 15 workplaces in Denmark, representing three occupational sectors (cleaning, manufacturing, and transportation), and white-collar workers with administrative tasks in the same workplaces. The workplaces were selected to obtain a sufficient dispersion in physical activities at work. Data collection occurred from April 2012 to May 2014. During baseline, data collection consisted of a web-based questionnaire, a health examination, and device-worn measurements of physical behaviors. During follow-up, data on self-reported sick leave were collected every fourth week over one year (14 waves in total) using text messages.

We invited 2107 workers through a screening questionnaire, 1119 were interested in participating, and 1087 entered the study at baseline [26]. Of those, 789 workers were included after providing valid data of physical behavior at baseline and sick leave during follow-up (described in detail below). Workers who participated reported similar characteristics in various demographic, occupational, and pain-related factors compared to those not participating [26].

All participants provided their written informed consent prior to participation. The present study was conducted according to the Declaration of Helsinki, approved by the Danish Data Protection Agency, and evaluated by the Regional Ethics Committee in Copenhagen, Denmark (H-2-2012-011).

### 2.2. Assessment of Physical Behavior

Physical behaviors were assessed at baseline using accelerometers (Actigraph GTX+, Actigraph, Pensacola, FL, USA) attached to the thigh and trunk for up to 5 working days, as described in previous studies on the DPHACTO cohort [21,27], with most recordings starting on Tuesdays and Wednesdays. Using the validated Acti4 software [28,29], time spent in sitting, standing, light physical activity (LIPA: moving and slow walking) and moderate- to- vigorous physical activity (MVPA: fast walking, stair walking, running, and cycling) were determined at work and leisure (i.e., non-work, excluding sleep) during each measurement day and averaged across days. The percentage of time spent in each behavior at work and leisure was then determined prior to further analyses.

A paper diary was used to note time stamps indicating periods of work and non-work, as well as periods of not wearing the accelerometers. Non-wear time was determined offline using an automatic algorithm (i.e., >90 min with 0 accelerometer counts) combined with the diary reports and visual inspection.

Valid accelerometer records had to contain at least one complete day with valid data at work and leisure. The criteria for valid work and leisure periods were at least 4 h per day of accelerometer wear time, or 75% of the average wear time across days for the individual.

### 2.3. Sick Leave Due to Musculoskeletal Pain

We used self-reported information on sick leave due to the musculoskeletal pain that was assessed using text messages (SMS), administered every fourth week over 1 year [4]. The number of days on sick leave due to MSP was assessed using a single item from the validated Outcome Evaluation Questionnaire [30]: “Within the past month, how many days have you been absent from work due to pain in muscles or joints?” The response scale ranged from 0 days to 28 days. We used latent class growth analysis to identify four distinct trajectories of sick leave due to MSP, as described previously [4]. The four trajectories were based on the amount of sick-leave days at baseline and to what extent the number of days on sick leave followed an increasing or decreasing trajectory over one year (i.e., “no sick leave”, “few days—increasing trajectory”, “some days—decreasing trajectory”, and “some days—increasing trajectory”). Workers with less than two responses to text messages at follow-up were excluded from the analysis. We used the pre-identified trajectories as a categorical outcome in the present study.

### 2.4. Covariates

Based on previous research on factors that could influence the association between physical behavior and sick leave, we included several covariates in this study. We used company records to assess age (years) and sex (male or female). Smoking habits were assessed by self-reported responses to the question, “do you smoke?”, using four response categories, which were recoded into smokers (daily) and non-smokers (occasionally, formerly, or never). We measured height and weight to calculate body mass index (BMI). MVPA during leisure time was assessed using accelerometer data, combining time spent fast walking, running, cycling, and stair walking. Lifting and carrying at work were measured using a self-report: “How much of your working time are you carrying or lifting?” with a scale of 1–6 (never, rarely, a quarter of the time, half of the time, three-quarters of the time, and almost all the time). We measured musculoskeletal pain in different body sites using a numeric rating scale from 0 (no pain) to 10 (worst pain imaginable), asking about the worst pain during the previous three months. For each pain site (neck/shoulder, elbow, lower back, hip, knee, and feet/ankle), a pain intensity score of at least 3 was used to describe the occurrence of pain.

### 2.5. Statistical Analyses

Time spent in sitting, standing, LIPA, and MVPA was transformed to isometric log-ratios (ILRs) according to the CoDA approach [23,31]. The composition of physical behaviors at work was expressed in four unique sets (one for each behavior as the numerator) of three ILR coordinates expressing time spent in each behavior relative to the other behaviors [23]. For example, sitting relative to non-sitting (ILR1), standing relative to physical activity (ILR2), and LIPA relative to MVPA (ILR3). For MVPA during leisure, data were transformed into one ILR reflecting a two-part composition expressing MVPA relative to non-MVPA.

We constructed multinomial regression models to determine the association between the first ILR in each compositional set and sick leave due to MSP (four categories, referencing no sick leave), with adjustment for the remaining composition (i.e., ILR2 and ILR3 in each set). Models were then constructed with additional adjustment for age, gender, BMI, smoking habits, and MVPA during leisure. Workers with missing data on covariates were excluded.

Three sensitivity analyses were done. First, the primary models were re-run on blue-collar workers only, excluding white-collar workers. Second, the primary models were re-run with additional adjustment for baseline sick leave. Due to a large number of zeroes, days on sick leave the past month were dichotomized as “0 days” and “1 day or more”. Third, the primary models were re-run with adjustment for the duration of lifting and carrying at work, as a proxy for high biomechanical load.

Firth’s bias correction was applied to the primary models to account for the small number of workers in some of the sick-leave groups [32]. For each model, we obtained the odds ratio (OR) with 95% confidence intervals (CIs). *p*-Values < 0.05 were considered statistically significant.

To ease interpretation of the effect sizes obtained from models on transformed data (ILRs), isotemporal substitution was performed. From the mean composition, we reallocated time (−60 min to 60 min) in each behavior by proportionally increasing or decreasing time in the other behaviors, recalculating compositions (ILRs), and obtaining estimated ORs from predicted probabilities [33,34].

## 3. Results

### 3.1. Characteristics of the Study Sample

The average age in the study sample was 45 (SD 10) years, 55% were men, and 81% had blue-collar jobs. BMI had an average of 27 kg/m^2^ (SD 5), and 14.5% (SD 3.5) of leisure time was spent in MVPA. The average worker reported 6 (SD 20) days on sick leave in total during 1-year follow-up. The four sick leave trajectories due to MSP differed in total days on sick leave over 1 year, ranging from less than one day (i.e., no sick leave) to almost 100 days over 1 year (i.e., some days—increasing trajectory). Descriptive data of the four trajectories are shown in Table 1.

### 3.2. Physical Behaviors at Work

**Figure 1:**
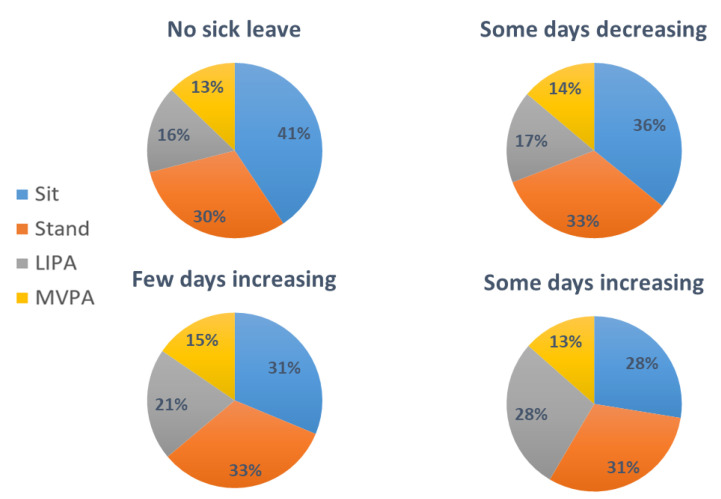
Mean work-time composition (% time) of physical behaviors (i.e., sitting, standing, LIPA (light physical activity), and MVPA (moderate-to-vigorous physical activity)) in each sick-leave trajectory.

On average, workers spent 38% (SD 24) of their working time sitting (blue-collar 32%, white-collar 65%), 31% (SD 15) standing (blue-collar 33%, white-collar 21%), 17% (SD 11) in LIPA (blue-collar 20%, white-collar 6%), and 13% (SD 7) in MVPA (blue-collar 15%, white-collar 8%). Figure 1 shows the means of percentage of time in physical behaviors at work in the four trajectories of sick leave due to MSP.

### 3.3. Association between Work-Time Compositions of Behavior and Sick Leave Due to MSP

**Figure 2:**
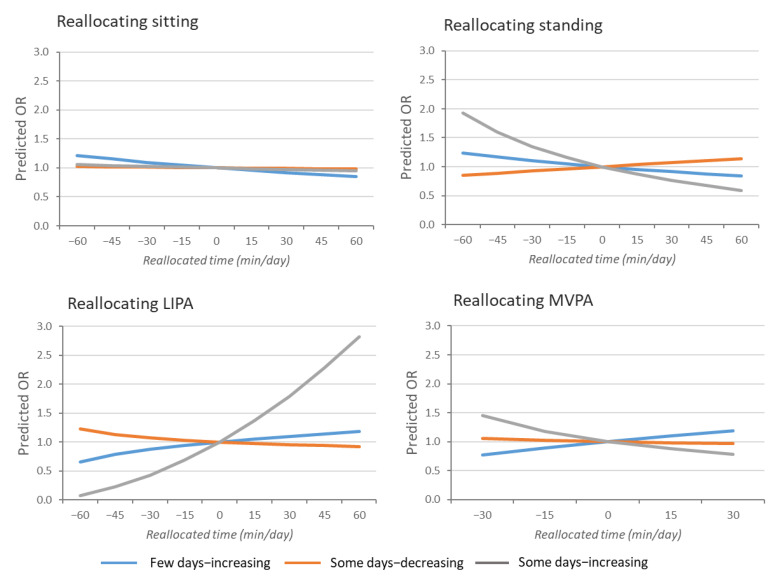
Predicted odds ratios (ORs) of sick leave trajectories when reallocating time in each behavior at work (sitting, standing, LIPA, and MVPA) to all other behaviors. The trajectory with no sick leave was used as reference.

More sitting time at work was significantly (adjusted *p* = 0.004) associated with a reduced likelihood of belonging to the trajectory of few days—increasing sick leave due to MSP compared to the trajectory with no sick leave (Table 2). Reallocations indicated that increasing sitting time at work by 60 min/day, by proportionally decreasing time in the other behaviors, predicted a 15% (OR 0.85) reduced likelihood of belonging to the trajectory of few days—increasing sick leave due to MSP (Figure 2).

More time in LIPA at work was significantly (adjusted *p* = 0.009) associated with an increased likelihood of belonging to the trajectory with some days—increasing sick leave due to MSP compared with the trajectory with no sick leave (Table 2). Reallocating 60 min to LIPA from other behaviors at work predicted a 180% (OR 2.8) increase in likelihood of some days—increasing sick leave due to MSP (Figure 2).

Standing time and MVPA at work did not show any significant association with sick leave trajectories (negative estimates, adjusted *p* > 0.05). None of the physical behaviors were significantly associated with the trajectory with some days—decreasing sick leave due to MSP (*p* > 0.05).

### 3.4. Sensitivity Analyses

Three sensitivity analyses were done to test the robustness of the primary model results (Table 3). First, the models only on blue-collar workers (n = 636) yielded similar associations between work-time compositions and sick-leave trajectories compared to the primary models, although the negative association between sitting and the trajectory with few days—increasing sick leave became borderline non-significant (OR = 0.78, 95% CIs 0.60–1.01, *p* = 0.058). Second, models with additional adjustment for sick leave at baseline did not change the estimates or significance compared to the primary models. Third, models with additional adjustment for lifting and carrying at work showed similar estimates and significance as the primary models, although the negative estimate for standing and some days—increasing sick leave became slightly stronger and more significant (OR = 0.20, 95% CI 0.05–0.80, *p* = 0.023).

## 4. Discussion

To the best of our knowledge, this is the first longitudinal study focusing on associations between compositions of physical behavior at work and sick leave due to MSP. The main strengths of this study are the device-worn measurements of exposure combined with frequent measurements of cause-specific sick leave. In addition, we used CoDA to address the compositional nature of time-use exposure data in our statistical models. This allowed us to obtain trustworthy estimates of both exposure and outcome and to determine the effects of work-time compositions, including multiple behaviors.

Our main findings indicated that work-time compositions with more sitting were associated with a lower risk of an increasing trajectory of sick leave due to MSP over one year, while compositions with more LIPA were associated with a higher risk. On average, the workers in this sample spent 38% of their working time sitting, 31% standing, 17% in LIPA, and 13% in MVPA. We estimated that increasing sitting time at work by 60 min—at the expense of standing, LIPA, and MVPA—significantly reduced the likelihood of the trajectory “few days—increasing sick leave due to MSP” (OR 0.85) compared to the “no sick leave” trajectory. This result could be explained by the population of mainly blue-collar workers who are often exposed to physically demanding tasks known to be associated with MSP and sick leave in previous studies [35,36,37]. Thus, sitting may be needed to allow rest and recovery, and prevent aggravated symptoms that contribute to sick-leave spells [38]. This would corroborate previous prospective studies using device-worn measurements indicating a negative association between sitting time and MSP in blue-collar workers [12]. To account for the possibility of this association merely occurring because of sitting being inversely related to physical activity, we adjusted for relative time spent in standing, LIPA, and MVPA, along with other relevant covariates.

Moreover, our models estimated that a 60 min increase in LIPA at work significantly increased the likelihood of the trajectory “some days—increasing sick leave” (OR 2.8), compared to the “no sick leave” trajectory. This would indicate that prolonged exposure to slow-walking and other low-intensity movements at work is associated with a higher risk of increasing the number of days on sick leave due to MSP over one year. This may be explained by high energy demands and activities not providing sufficient recovery, although this needs further investigation. Still, results suggest that interventions reducing LIPA at work by increasing sitting may be effective for the prevention of sickness absence due to pain, at least in blue-collar jobs.

It is important to note that exposure patterns of physical behaviors at work could be largely different between occupational groups, which could modify the associations with sick leave due to pain. We found that the time-use composition of physical behaviors at work in white-collar workers was predominated by sitting time, while the blue-collar workers had relatively less time in sitting and more time in LIPA and MVPA. To address this issue, we conducted a sensitivity analysis in blue-collar workers. We found similar associations compared to the primary analysis in the whole sample, although the association between sitting time and “few days—increasing sick leave” became slightly less significant (*p* = 0.058), likely due to the smaller sample. This indicates that the observed associations were not confounded by different compositions of physical behaviors at work between blue and white-collar workers. However, as our estimates largely pertain to blue-collar workers, inferences to other occupational groups should be made with caution. Additionally, relationships between physical behavior at work and sick leave may differ between job types due to various work tasks and biomechanical loads. Unfortunately, our study was restricted to cleaning, manufacturing, and transportation sectors, and the sample size did not justify specific analyses by occupation. Thus, future studies should preferably conduct occupation specific analyses and disentangle the effects of physical behavior, work tasks, and biomechanical loads on pain and sick leave. We also recommend looking further into moderation by several factors like age, gender, socioeconomic position, individual health status, and physical capacity.

We did not find any statistically significant associations for time spent in standing and MVPA in the primary models, which may be due to several reasons, such as insufficient dispersion of these behaviors between workers and the limited sample size, resulting in wider confidence intervals. However, standing time was significantly negatively associated with the trajectory “some days—increasing sick leave” in the model with additional adjustment for lifting and carrying at work. This may be a chance finding and should be interpreted with caution, but it may suggest that replacing LIPA with standing time is beneficial for sick leave due to pain.

Overall, our findings add to previous evidence on physical behavior and MSP in workers [12,27,39] and suggest that sitting at work may prevent sick leave due MSP among blue-collar workers, while low-intensity physical activity may increase the risks.

### Methodological Discussion

The limited number of workers in the trajectories with “some days—decreasing” (n = 18) and “some days—increasing” (n = 17) sick leave due to MSP is a limitation leading to less-certain estimates and limited statistical power. Although we addressed this issue by adjusting model estimates using Firth’s bias correction [32], a larger sample would likely have led to more accurate results. Moreover, the sample size did not allow stratified analyses by occupation, which may modify associations between work-time compositions and sick leave. Still, we performed a sensitivity analysis only on blue-collar workers and found no critical change in the effect sizes compared to the primary analysis that also included workers with office-based jobs. However, as the predominance of blue-collar workers in the study hampers the generalizability to other occupational groups, we recommend future studies to address physical behaviors at work and sick leave due to MSP in larger cohorts enabling stratified analyses.

We assessed sick leave due to pain without distinguishing different diagnoses, localizations, or severities of MSP. It is possible that the relationship between physical behavior at work and sick leave due to pain is modified by these factors.

There is also the possibility of reversed causality with sick leave affecting behaviors at work. However, we addressed this possible issue by adjusting for the occurrence of sick leave at baseline. This adjustment did not alter the estimates or significance of the associations, which indicates that reversed causality was not a critical issue.

We addressed several factors that could potentially confound associations between physical behaviors and sick leave, such as age, gender, BMI, smoking habits, and physical activity during leisure. However, we cannot preclude residual confounding by unmeasured factors.

## 5. Conclusions

We found that compositions with more sitting relative to the other behaviors had lower odds for increasing trajectories of sick leave due to musculoskeletal pain, while compositions with more light physical activity had higher odds. This may have implications for the prevention of pain-related sick leave in blue-collar workers.

## Figures and Tables

**Table 1:** Descriptive data in the study sample stratified by sick leave trajectory due to MSP.

Variables		No Days	Few Days—Increasing	Some Days—Decreasing	Some Days—Increasing
	n	(n = 600)	(n = 154)	(n = 18)	(n = 17)
Age, mean (SD)	789	44.5 (10.1)	44.2 (9.4)	46.9 (7.5)	48.6 (11.7)
Sex	789				
Men, n (%)	430	336 (56)	79 (51)	7 (39)	8 (47)
Women, n (%)	359	264 (44)	75 (49)	11 (61)	9 (53)
Occupational class	789				
White-collar workers, n (%)	153	139 (23)	12 (8)	2 (11)	0 (0)
Blue-collar workers, n (%)	636	461 (77)	142 (92)	16 (89)	17 (100)
Smoking	772				
Smokers, n (%)	160	102 (17)	46 (31)	5 (28)	7 (47)
Non-smokers, n (%)	612	488 (83)	103 (69)	13 (72)	8 (53)
BMI (kg/m^2^), mean (SD)	773	27.2 (4.7)	27.7 (4.8)	30.0 (5.4)	26.0 (6.1)
Percent MVPA leisure, mean (SD)	775	14.6 (3.7)	14.2 (3.3)	13.4 (2.3)	14.9 (2.3)
Lifting and carrying at work ^a^, mean (SD)	783	3.0 (1.5)	3.4 (1.6)	3.2 (1.3)	4.5 (1.3)
Total sick leave days, mean (SD)	789	0.4 (1.0)	11.4 (8.3)	51.7 (29.2)	94.7 (82.3)
Baseline sick leave days, mean (SD)	789	0.1 (0.4)	0.8 (2.3)	5.2 (7.9)	2.0 (3.3)
Baseline musculoskeletal pain, n (%)					
Neck/shoulder	789	305 (50.8)	94 (61.0)	12 (66.7)	11 (64.7)
Elbow	789	74 (12.3)	32 (20.8)	6 (33.3)	3 (17.6)
Hand/wrist	789	120 (20.0)	52 (33.8)	5 (27.8)	8 (47.1)
Lower back	789	291 (48.5)	101 (65.6)	12 (66.7)	15 (88.2)
Hip	789	81 (13.5)	49 (31.8)	9 (50.0)	7 (41.2)
Knee	789	179 (29.8)	70 (45.5)	8 (44.4)	6 (35.3)
Foot/ankle	789	134 (22.3)	51 (33.1)	6 (33.3)	5 (29.4)

Abbreviations: BMI, body mass index; MVPA, moderate-to-vigorous physical activity. ^a^ The response scale ranges from 1 (never) to 6 (almost all the time).

**Table 2:** Adjusted association between work-time compositions of physical behavior and sick leave due to musculoskeletal pain (reference: no sick leave; n = 571) in 743 workers. Physical behavior data are expressed in isometric log-ratios (ILRs).

	1-Year Trajectories of Sick Leave Due to Musculoskeletal Pain (Outcome)					
	Few Days—Increasing		Some Days—Decreasing		Some Days—Increasing	
	(n = 140)		(n = 18)		(n = 14)	
Predictors	OR	95% CI	OR	95% CI	OR	95% CI
Sitting/non-sitting	**0.71**	**0.56–0.90**	0.99	0.56–1.75	0.96	0.47–1.96
Standing/non-standing	0.73	0.48–1.11	1.42	0.50–4.03	0.34	0.10–1.16
LIPA/non-LIPA	1.34	0.77–2.32	0.85	0.22–3.32	**6.23**	**1.58–24.55**
MVPA/non-MVPA	1.45	0.85–2.47	0.84	0.21–3.38	0.49	0.12–1.93

Models are adjusted for age, sex, body mass index, smoking habits, MVPA during leisure, and the remaining composition of behavior (i.e., ILR2 and ILR3). Significant (*p* < 0.05) associations are in bold face. Abbreviations: LIPA, light physical activity; MVPA, moderate-to-vigorous physical activity.

**Table 3:** Sensitivity analyses. Adjusted association between work-time compositions of physical behaviors and sick leave due to musculoskeletal pain. No sick leave was used as reference. Physical behavior data are expressed in isometric log-ratios (ILRs).

	1-Year Trajectories of Sick Leave Due to Musculoskeletal Pain (Outcome)					
	Few Days—Increasing		Some Days—Decreasing		Some Days—Increasing	
Predictors	OR	95% CI	OR	95% CI	OR	95% CI
Models only on blue-collar workers (n = 636)						
Sitting/non-sitting	0.78	0.60–1.01	1.21	0.66–2.22	1.25	0.63–2.47
Standing/non-standing	0.70	0.45–1.09	1.34	0.44–4.06	0.38	0.11–1.26
LIPA/non-LIPA	1.29	0.71–2.35	0.79	0.18–3.57	5.05	1.24–20.57
MVPA/non-MVPA	1.37	0.79–2.39	0.75	0.18–3.11	0.46	0.12–1.83
Models adjusting for baseline sick leave (n = 743)						
Sitting/non-sitting	0.72	0.57–0.92	1.10	0.56–2.16	0.95	0.45 -2.03
Standing/non-standing	0.74	0.48–1.14	1.40	0.45–4.32	0.37	0.10–1.30
LIPA/non-LIPA	0.74	0.48–1.14	0.90	0.19–4.14	6.00	1.40–25.81
MVPA/non-MVPA	1.36	0.79–2.33	0.72	0.16–3.35	0.47	0.11 -2.07
Models adjusting for lifting and carrying at work (n = 741)						
Sitting/non-sitting	0.72	0.56–0.53	0.98	0.53–1.82	1.62	0.74–3.52
Standing/non-standing	0.73	0.48–1.12	1.45	0.50–4.19	0.20	0.05–1.80
LIPA/non-LIPA	1.29	0.74–2.25	0.84	0.21–3.28	5.74	1.24–26.44
MVPA/non-MVPA	1.47	0.86–2.50	0.84	0.21–3.39	0.54	0.13–2.20

All models are adjusted for age, sex, body mass index, smoking habits, MVPA during leisure, and the remaining composition of behavior (i.e., ILR2 and ILR3). Abbreviations: LIPA, light physical activity; MVPA, moderate-to-vigorous physical activity.

## Data Availability

The data presented in this study are available on request (A.H: aho@nfa.dk).
